# Bio-Functional Sperm Parameters: Does Age Matter?

**DOI:** 10.3389/fendo.2020.558374

**Published:** 2020-12-11

**Authors:** Rosita A. Condorelli, Sandro La Vignera, Federica Barbagallo, Angela Alamo, Laura M. Mongioì, Rossella Cannarella, Antonio Aversa, Aldo E. Calogero

**Affiliations:** ^1^ Department of Clinical and Experimental Medicine, University of Catania, Catania, Italy; ^2^ Department of Experimental and Clinical Medicine, Magna Graecia University of Catanzaro, Catanzaro, Italy

**Keywords:** spermatozoa, sperm DNA fragmentation, sperm apoptosis, male age, sperm mitochondrial membrane potential, sperm lipid peroxidation, alive spermatozoa

## Abstract

The evaluation of biofunctional sperm parameters can explain some cases of idiopathic male infertility. Among these, sperm DNA fragmentation (fDNA) is the most studied biofunctional sperm parameter. Mitochondrial membrane potential (MMP) correlates positively with sperm motility, the evaluation of sperm apoptosis by flow cytometry allows us to identify a population of spermatozoa not recognizable at the optical microscopy and finally, lipid peroxidation (LP) and mitochondrial superoxide levels measurements are rational oxidative stress indices. Male age seems to affect sperm concentration and sperm fDNA. For these reasons, this study was undertaken to evaluate the correlation, if any, between male age and biofunctional sperm parameters evaluating their possible impact on fDNA. To accomplish this, MMP, degree of chromatin compactness, sperm apoptosis/vitality, fDNA, LP, and mitochondrial superoxide levels were evaluated by flow cytometry in a cohort of 874 men. A significant negative correlation was found between age and the percentage of alive spermatozoa (r = -0.75, p < 0.05). The percentage of spermatozoa with low MMP (L-MMP) correlated positively with the percentage of spermatozoa with abnormal chromatin compactness (r = 0.24, p < 0.05). Spermatozoa with abnormal chromatin compactness and L-MMP correlated negatively with the percentage of alive spermatozoa (r = 0.83, p < 0.05) and positively with spermatozoa with PS externalization (r = 0.13, p < 0.01). The percentage of alive spermatozoa correlated negatively with both the percentage of spermatozoa with PS externalization (r = 0.24, p < 0.01) and of the spermatozoa with fDNA (r = 0.10, p < 0.05). Spermatozoa with PS externalization correlated positively with the percentage of spermatozoa with fDNA (r = 0.09, p < 0.05). Spermatozoa with LP correlated positively with the percentage of spermatozoa with increased mitochondrial superoxide (r = 0.11, p < 0.01) In conclusion, these findings in a large number of men suggest that age, mitochondrial damage, and alteration of chromatin compactness could activate the apoptotic cascade which could result in an increased fDNA rate.

## Introduction

Biofunctional sperm parameters can alter conventional sperm parameters and can explain some cases of idiopathic male infertility (1).

Sperm DNA fragmentation (fDNA) is the most studied biofunctional sperm parameter to ensure normal fertilization, embryo development, and implantation (2). Physiologically, sperm DNA breaks up during spermiogenesis to allow chromatin reorganization. When DNA is complexed with protamine is very stable and it becomes resistant to digestive enzymes. McPherson and Longo observed the presence of damaged DNA strands and proposed that the breaks could be due to an incorrect replacement of histones with protamine (3). These cuts are then repaired by the topoisomerases II or, after fertilization, by the oocyte repair machinery. In this regard, the degree of chromatin compactness is another biofunctional sperm parameter that allows us to ascertain sperm damage due to DNA immaturity.

Among the causes of fDNA, some are reversible and the improvement of this parameter could be fundamental not only for natural pregnancies but also for patients requiring assisted reproductive techniques (ART) for their infertile condition. A recently published meta-analysis showed a negative impact of fDNA on ICSI outcomes (4). Other studies have shown that the percentage of sperm fDNA correlates with conventional sperm parameters, but this association is not very strong, indicating that sperm fDNA could independently predict “semen quality” (5).

Mitochondrial membrane potential (MMP) evaluation is another important marker of sperm mitochondrial function that correlates positively with sperm motility (6). The evaluation of sperm apoptosis/viability allows us to identify a population of spermatozoa that have already initiated the first steps of the apoptotic cascade not recognizable at the optical microscopy. Finally, lipid peroxidation (LP) and mitochondrial superoxide levels measurements are sensible oxidative stress indices, as previously described in systemic diseases, such as diabetes mellitus (7, 8). Nevertheless, oxidative membrane damage does not always cause significant intracellular response, according to the adaptation capacity of the organism that is influenced by different elements of susceptibility (e.g., previous antioxidant therapy) (9).

However, many of the studies so far cited do not consider the age of the male patients. This aspect is of fundamental relevance because couples are trying to conceive at an increasingly advanced age than in the past. The correlation between age and sperm fDNA has been well studied, however, some aspects remain controversial. Male age seems to affect significantly sperm parameters, such as sperm concentration and sperm fDNA (10). Men older than 40 years have a higher rate of sperm fDNA (sperm fDNA index of >10%) compared to men aged <40 years (11). A retrospective study (2013–2016 years) evaluated the correlation between sperm DNA integrity and male age in 654 infertile patients: men aged ≥35 years have increased sperm fDNA rate independently of conventional sperm parameters (12). Another study indicates a statistically significant increase only after 45 years of age (13). Kaarouch and colleagues reported increased fDNA, chromatin decondensation, and sperm aneuploidy rates in the group of men with advanced age (≥ 40 years) and that the paternal age influences ART outcomes, such as canceled embryo transfers, clinical pregnancy, and increased miscarriage (14). However, other studies have not confirmed the influence of age on sperm fDNA and in achieving pregnancy (15, 16).

Therefore, this study aimed to analyze the correlation between male age and biofunctional sperm parameters and to evaluate, for the first time, their possible impact that can result in fDNA. To accomplish this, biofunctional sperm parameters (mitochondrial function, degree of chromatin compaction, cellular apoptosis, and oxidative stress) were evaluated in a large cohort of men aged 18 to 55 years to investigate possible intracellular pathways that may alter sperm fDNA.

## Patients and Methods

### Patient Selection

The study was conducted on 874 unselected men, aged between 18 and 55 years (35.1 ± 8.2 years). This study was approved by the Ethics Committee of the University Teaching Hospital of “Policlinico-Vittorio Emanuele”, University of Catania, (Catania, Italy). All methods were performed by following the more relevant guidelines (17, 18) and regulations. All participants were asked to sign their informed consent.

### Experimental Design

Each man enrolled in this study underwent to semen analysis. Semen samples were collected by masturbation after 3–5 days of sexual abstinence. After 30 min of liquefaction at 37°C, they were washed with PBS and immediately acquired by flow cytometry for the evaluation of biofunctional sperm parameters. The latter included: mitochondrial membrane potential (MMP) evaluation, degree of chromatin compactness, sperm apoptosis/vitality evaluation, fDNA assessment, lipid peroxidation (LP), and mitochondrial superoxide levels determination, as previously reported (7). The same procedures were undertaken at the same laboratory for these studies despite different populations being used (1, 7).

### Cytofluorimetric Analysis

Cytofluorimetric analysis was performed using flow cytometer CytoFLEX (Beckman Coulter Life Science, Milan) equipped with two argon lasers and six total fluorescence channels (four 488 nm and two 638 nm). We used the FL1 detectors for green (525 nm), FL2 for orange (585 nm), and FL3 for red (620 nm) fluorescence; 100,000 events (low velocity) were measured for each sample and analyzed by the software CytExpert 1.2.

### Evaluation of the Mitochondrial Membrane Potential

MMP was evaluated using the lipophilic probe 5,5’,6,6’-tetrachloro-1,1’,3,3’tetraethyl-benzimidazolylcarbocyanine iodide (JC-1, DBA s.r.l, Milan, Italy). An aliquot containing 1x10^6^/ml of spermatozoa was incubated with JC-1 in the dark, for 10 min, at a temperature of 37°C. At the end of the incubation period, the cells were washed in PBS and analyzed. JC-1 penetrates selectively in mitochondria existing in monomeric form, emitting at 527 nm (giving a green fluorescence for cells with low MMP (L-MMP), while after excitation at 490 nm and based on the membrane potential, JC-1 forms aggregates emitting at 590 nm (cells with normal or high MMP with a double fluorescence, green and orange). Negative control was obtained omitting fluorescence from the reaction mixture, while the addition of carbonyl cyanide 3-chlorophenylhydrazone can be used to confirm that the JC-1 response is sensitive to changes in membrane potential.

### Assessment of the Degree of Chromatin Compactness

An aliquot of 1x10^6^ spermatozoa was incubated with LPR DNA-Prep Reagent containing 0.1% potassium cyanate, 0.1% NaN3, non-ionic detergents, saline, and stabilizers (Beckman Coulter, IL, Milan, Italy) for cell membrane permeabilization, in the dark, at room temperature for 10 min and then further incubated with Stain DNA-Prep Reagent containing 50 µg/ml of propidium iodide (PI) (<0.5%), RNase A (4 KUnitz/ml), <0.1% NaN3, saline, and stabilizers (Beckman Coulter, IL, Milan, Italy) in the dark at room temperature. Flow cytometry analysis was performed after 30 min, by FL3 detector: the greater the fluorescence emitted, the greater the binding points inside the double helix indicate a worse chromatin compactness.

### Evaluation of Sperm Apoptosis/Vitality

The signal of early apoptosis is the phosphatidylserine (PS) externalization on the outer cell surface. The assessment of PS externalization was performed using annexin V, the protein that binds selectively to PS in the presence of calcium ions, FITC-labeled. When the cell membrane is also damaged the intracellular passage of the propidium iodide (PI) takes place. Therefore, marking simultaneously the cells with annexin V and PI, we distinguished three different cell populations: viable cells, cells in early apoptosis, and cells in late apoptosis. Staining with annexin V and PI was obtained using a commercially available kit (Annexin V-FITC Apoptosis, Sigma Chemical).

An aliquot containing 0.5 × 10^6^/ml was suspended in 0.5 ml of buffer containing 10 µl of annexin V-FITC and 20 µl of PI and incubated for 10 min in the dark. After incubation, the sample was analysed immediately by the detectors FL-1 (FITC) and FL3 (PI).

### Assessment of DNA Fragmentation

The evaluation of fDNA was performed by the TUNEL method. This uses the TdT (Terminal deoxynucleotidyl Transferase), an enzyme that polymerizes, at the level of DNA breaks, modified nucleotides conjugated to a fluorochrome. The TUNEL assay was performed by using a commercially available kit (Apoptosis Mebstain kit, Beckman Coulter, Milan). To obtain a negative control, TdT was omitted from the reaction mixture; the positive control was obtained pretreating spermatozoa (about 0.5 × 10^6^) with 1 mg/ml of deoxyribonuclease I, not containing RNAse, at 37°C for 60 min before staining. The reading was performed by flow cytometry using the FL1 detector.

### Evaluation of Lipid Peroxidation

The probe BODIPY (581/591) C11 was used for LP evaluation by flow cytometry. It is incorporated into cell membranes and responds to the attack of free oxygen radicals changing its spectrum of emission from red to green. This displacement of the emission is shown by the flow cytometer, which provides an estimate of the degree of peroxidation.

About 2 × 10^6^ of spermatozoa were incubated with 5 mM of the probe for 30 min in a final volume of 1 ml. After washing with PBS, flow cytometry analysis was conducted using the FL1 and FL2 detectors (19).

### Measurement of Mitochondrial Superoxide

Mitochondrial superoxide levels were detected by the MitoSOX red mitochondrial superoxide indicator (20). It penetrates the mitochondria and it is quickly oxidized by superoxide anion (not from other free radicals) becoming highly fluorescent with signal detection.

### Statistical Analysis

The Kolmogorov-Smirnov test was used to evaluate whether each variable had a Gaussian distribution. Non-parametric tests were then used because the variables were not normally distributed. Each variable was evaluated according to the quartiles of age, and the results are reported as medians and interquartile ranges. The Spearman correlation analysis was used to investigate the association between age and bio-functional sperm parameters and among all the bio-functional sperm parameters evaluated in this study. The software SPSS 23.0 for Windows (SPSS Inc., Chicago, USA) was used. A p-value lower than 0.05 was considered statistically significant.

## Results

The median and interquartile range of each biofunctional sperm parameter evaluated in the 874 men enrolled in this study are reported in **Table 1**.

**Table 1:** Bio-functional sperm parameters in all men studied.

Bio-functional sperm parameters	Values
Low mitochondrial membrane potential (%)	41.4 (41)
Chromatin compactness (%)	22.2 (10.8)
Alive spermatozoa (%)	69.0 (25.3)
Early apoptosis spermatozoa (%)	1.3 (2.3)
Late apoptosis spermatozoa (%)	3.6 (8.4)
Sperm DNA fragmentation (%)	2.0 (3.8)
Lipid peroxidation (%)	2.2 (2.9)
Sperm mitochondrial superoxide (%)	43.9 (41.3)

Results are shown as median and interquartile ranges in parentheses.

**Figure 1:**
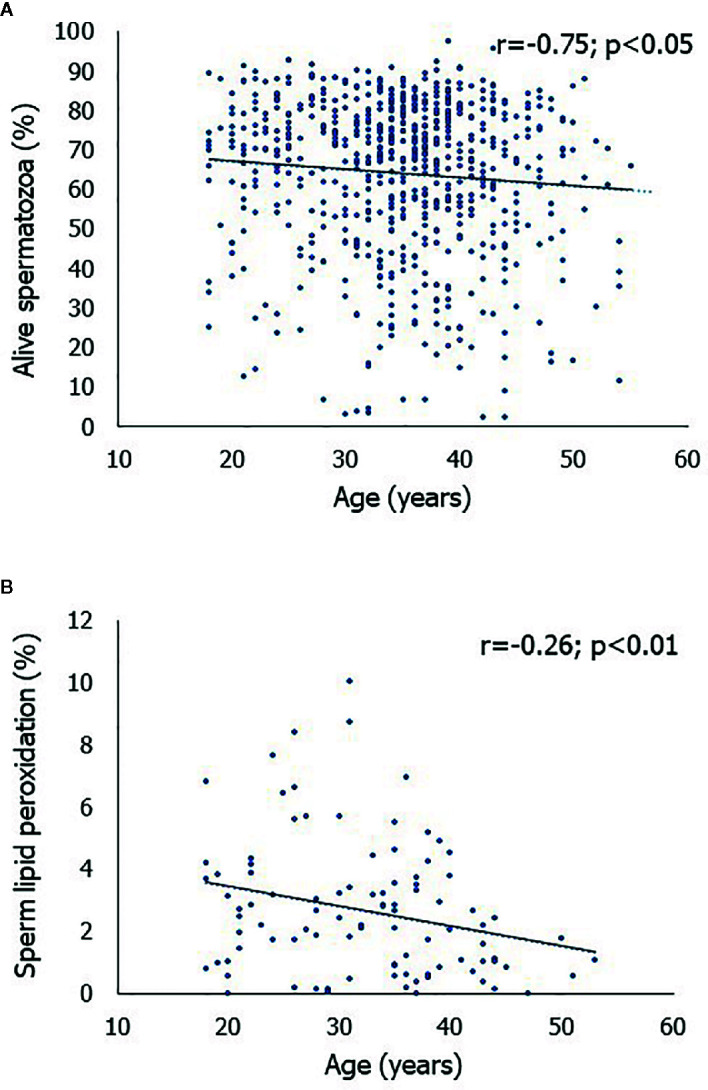
Scatterplot of the correlations between age of the men enrolled in this study and the percentage of alive spermatozoa **(A)** and between age and the percentage of spermatozoa with lipid peroxidation **(B)**.

A significantly negative correlation was found between the age of the men and the percentage of alive spermatozoa (r = -0.75, p < 0.05) (**Figure 1A**). Accordingly, we found that the first quartile of the percentage of alive spermatozoa based on the men’s age (min, 2.9; max, 92.9) was statistically significantly lower (p < 0.01) than the fourth quartile (min, 2.4; max, 95.8). Also, we found that age correlated negatively with the percentage of spermatozoa with LP (r = -0.26, p < 0.01) (**Figure 1B**).

**Table 2** shows the significant correlations among the biofunctional sperm parameters. The percentage of spermatozoa with L-MMP correlated positively with the percentage of spermatozoa with abnormal chromatin compactness and with spermatozoa with PS externalization. A significant negative correlation was found between spermatozoa with L-MMP and the percentage of alive spermatozoa. Spermatozoa with abnormal chromatin compactness correlated negatively with the percentage of alive spermatozoa and positively with the percentage of spermatozoa with PS externalization. The percentage of alive spermatozoa correlated negatively with both the percentage of spermatozoa with PS externalization and in late apoptosis and of the spermatozoa with fDNA. Spermatozoa with PS externalization correlated positively with the percentage of spermatozoa with fDNA. Spermatozoa in late apoptosis correlated positively with the percentage of spermatozoa with PS and with fDNA and negatively with alive spermatozoa. Spermatozoa with LP correlated positively with the percentage of spermatozoa with increased mitochondrial superoxide. Finally, all the other correlations were not statistically significant (data are not shown).

**Table 2:** Spearman correlation analyses among bio-functional sperm parameters.

Parameter 1	Parameter 2	Correlation coefficient
Spermatozoa with low mitochondrial membrane potential (%)	Spermatozoa with abnormal chromatin compactness (%)	r = 0.24, p < 0.0001(n = 723)
	Alive spermatozoa (%)	r = -0.82, p < 0.05 (n = 696)
	Spermatozoa with phosphatidylserine externalization (%)	r = 0.09, p < 0.05(n = 696)
Spermatozoa with abnormal chromatin compactness (%)	Alive spermatozoa (%)	r = -0.83, p < 0.05 (n = 696)
	Spermatozoa with phosphatidylserine externalization (%)	r = 0.13, p < 0.001(n = 696)
Alive spermatozoa (%)	Spermatozoa with phosphatidylserine externalization (%)	r = -0.24, p < 0.01 (n = 696)
	Spermatozoa with fragmented DNA (%)	r = -0.10, p < 0.01 (n = 813)
Spermatozoa with phosphatidylserine externalization (%)	Spermatozoa with fragmented DNA (%)	r = 0.09, p < 0.005 (n = 813)
Late apoptosis spermatozoa (%)	Alive spermatozoa (%)	r = -0.36, p < 0.001 (n = 695)
	Spermatozoa with phosphatidylserine externalization (%)	r = 0.52, p < 0.001(n = 695)
	Spermatozoa with fragmented DNA (%)	r = 0.52, p < 0.001 (n = 695)
Spermatozoa with lipid peroxidation (%)	Spermatozoa with increased mitochondrial superoxide (%)	r = 0.11, p < 0.01 (n = 656)

The number of men who entered into the analysis are shown in parentheses; p, p-value.

## Discussion

The results of this study confirmed that advancing the male age is associated with decreased sperm viability. At the same time, the percentage of alive spermatozoa correlated negatively with the percentage of spermatozoa in early apoptosis and in late apoptosis, as predictable, and with fDNA rate. Furthermore, early apoptosis indicated as the percentage of spermatozoa with PS externalization showed a direct correlation with the rate of fDNA. These findings lead us to speculate that sperm apoptosis could be the source of fDNA and that age is also involved in this phenomenon, as we will discuss below.

In addition, the percentage of spermatozoa in early apoptosis was directly correlated with an alteration of sperm chromatin compactness and MMP, whereas no correlation was found between mitochondrial function or chromatin damage and fDNA. Therefore, both abnormal chromatin compactness and L-MMP could contribute to increasing sperm apoptosis rate and consequently the rate of fDNA. The increased percentage of spermatozoa with L-MMP is related to an increased rate of spermatozoa with abnormal chromatin compactness, indicating that mitochondrial damage can alter sperm DNA even without fragmenting it. A recent study conducted on patients with diabetes mellitus (DM) showed that type 1 DM damages the mitochondrial function and impairs sperm motility but has no effects on fDNA, whereas type 2 DM with its inflammatory conditions and the increased oxidative stress leads to reduced sperm viability and an increase in the rate of sperm fDNA rate (7). Other studies on these biofunctional sperm parameters have shown that fDNA occurs only if sperm apoptosis is triggered first (21–23). This condition is confirmed even after a specific treatment that improves the MMP without acting on sperm apoptosis and fDNA (24) and not only in spermatozoa but also in somatic cells, such as epithelial cells (25). Recent evidence confirms a role of PS in fertilization and in sperm:egg fusion (26). In addition, the apoptosis rate must probably exceed a given threshold to trigger fDNA because low percentages of spermatozoa in early apoptosis do not always end up with DNA fragmentation. This suggests that apoptosis is the trigger and not the consequence of fDNA. Furthermore, the increase in the number of spermatozoa with abnormal chromatin compactness does not directly correlate with fDNA. This can be explained by the fact that another mechanism, such as the apoptotic cascade, must be activated to induce DNA fragmentation.

Our study showed that age is associated with a decreased percentage of viable spermatozoa. The latter correlated negatively with fDNA; this is supported by other studies that have reported similar observations without however investigating its mechanism (10, 27). Muratori and colleagues reported also similar data using a different experimental design and methods to assess the degree of the sperm chromatin immaturity (evaluated by an excess of residual histones) and sperm apoptosis (evaluated by caspase activity and cleaved polyADP-ribose polymerase) (28).

The effects of aging on the decline of conventional sperm parameters (motility, morphology, and concentration) over time seem to be related to an alteration of steroidogenesis and increased oxidative stress (29). Advanced age is associated with epidemiological changes of the main systemic diseases which in turn can contribute to the deterioration of the semen quality. On the other hand, it is known that alterations of testicular function are often overlooked in aging because, due to an erroneous belief, testicular dysfunctions are not taken into account despite their great relevance for the serious systemic consequences. It is widely demonstrated that poor semen quality is the mirror of low quality of life and a reduced survival rate, as shown by studies analyzing the comorbidity indices (30). Alterations of sperm parameters can reflect a progressive dysfunction of Sertoli cells, which, by paracrine action, can alter the function of Leydig cells with consequent hypotestosteronemia (31). It is therefore time to consider that male hypogonadism may initially begin with an isolated Sertoli cell dysfunction that manifests as low sperm quality (32).

The present study did not show a correlation between age and increased oxidative stress indices (LP and mitochondrial superoxide levels). We used BODIPY (581/591) C11 as a sensitive marker of membrane LP by flow cytometry, regardless of whether it is activated by superoxide anion, or any other type of oxygen free radical. The positive correlation between LP and mitochondrial superoxide confirms the sensitivity of this assay. We found a statistically significant improvement in LP with advancing age. This finding seems to be in contrast with data reported elsewhere (29). However, it is necessary to take into account the great intra and interindividual variability of these parameters also in consideration of confounding factors, such as, for example, the use of supplements with antioxidant properties so widespread in our society (33). Furthermore, it should be noted that men enrolled in this study had an average age of 35.1 ± 8.2 years with an age range of 18-55 years, therefore younger than the studies that showed an increase in oxidative stress over time (29). The evaluation of the biofunctional sperm parameters as a marker of aging can be a very useful tool in the clinical practice for two reasons. First, to intercept the intracellular modifications that explain the progression of cell damage using a multi-parametric system and, secondly, to identify the altered sperm parameter to prescribe the most appropriate treatment (prokinetics, antioxidants, etc.) and evaluate the response regarding the improvement of the sperm parameter altered even before the clinical response becomes evident.

Finally, the results of this study add an important element in the pathophysiology of DNA damage. The advancing age of the male patient correlating with the increased apoptosis rate, which damages later the other biofunctional sperm parameters (mitochondrial damage and alteration of chromatin compactness), could increase in fDNA. Thus, the results of this study suggest evaluating fDNA to evaluate sperm fertilizing capability, especially in those couples undergoing ART cycles, as also suggested by specific guidelines (34). We suggest also to evaluate the other biofunctional sperm parameters since if on the one hand, they contribute to increasing the apoptosis rate, they can however already cause damage to the conventional sperm parameters as previously reported (1). Some altered parameters, such as MMP for example, if identified on time, are reversible and respond to a targeted treatment to avoid the consequent onset of apoptosis. Further studies are needed to understand the mechanisms and specific treatment to use in this category of patients.

In conclusion, the results of this study suggest that it is very important to take into account the contemporary evaluation of the biofunctional sperm parameters described. This to allow us to grasp subclinical elements and nuances that would go unnoticed through the conventional seminal fluid analysis or the evaluation of single biofunctional sperm parameter. Adult men should maintain a good quality of the conventional and biofunctional sperm parameters even if they do not have reproductive aims as a test of adequate functionality of the hypothalamic-pituitary-testicular axis and therefore of general health.

## Data Availability Statement

The datasets presented in this article are not readily available because of patient confidentiality and participant privacy. Requests to access the datasets should be directed to Rosita A. Condorelli: rosita.condorelli@unict.it

## Ethics Statement

The studies involving human participants were reviewed and approved by Division of Endocrinology “Policlinico G. Rodolico” Teaching Hospital–Catania University. The patients/participants provided their written informed consent to participate in this study.

## Author Contributions

RC and SV: conceptualization and draft preparation. FB, AA, and LM: methods. AA, RC and AC: revision of the text. All authors contributed to the article and approved the submitted version.

## Conflict of Interest

The authors declare that the research was conducted in the absence of any commercial or financial relationships that could be construed as a potential conflict of interest.
